# Musical experience influences socio-emotional functioning in behavioural variant frontotemporal dementia

**DOI:** 10.3389/fneur.2024.1341661

**Published:** 2024-01-24

**Authors:** Jochum J. van’t Hooft, Elia Benhamou, Claudia Albero Herreros, Jessica Jiang, Benjamin Levett, Lucy B. Core, Mai-Carmen Requena-Komuro, Chris J. D. Hardy, Betty M. Tijms, Yolande A. L. Pijnenburg, Jason D. Warren

**Affiliations:** ^1^Dementia Research Centre, UCL Queen Square Institute of Neurology, University College London, London, United Kingdom; ^2^Alzheimer Centre Amsterdam, Amsterdam UMC, Vrije Universiteit Amsterdam, Amsterdam, Netherlands; ^3^Amsterdam Neuroscience—Neurodegeneration, Amsterdam, Netherlands

**Keywords:** musical training, musician, music, frontotemporal dementia, social cognition, behaviour

## Abstract

**Objectives:**

On phenotypic and neuroanatomical grounds, music exposure might potentially affect the clinical expression of behavioural variant frontotemporal dementia (bvFTD). However, this has not been clarified.

**Methods:**

14 consecutive patients with bvFTD fulfilling consensus diagnostic criteria were recruited via a specialist cognitive clinic. Earlier life musical experience, current musical listening habits and general socio-emotional behaviours were scored using a bespoke semi-quantitative musical survey and standardised functional scales, completed with the assistance of patients’ primary caregivers. Associations of musical scores with behavioural scales were assessed using a linear regression model adjusted for age, sex, educational attainment and level of executive and general cognitive impairment.

**Results:**

Greater earlier life musical experience was associated with significantly lower Cambridge Behavioural Inventory (Revised) scores (β ± SE = −17.2 ± 5.2; *p* = 0.01) and higher Modified Interpersonal Reactivity Index (MIRI) perspective-taking scores (β ± SE = 2.8 ± 1.1; *p* = 0.03), after adjusting for general cognitive ability. Number of hours each week currently spent listening to music was associated with higher MIRI empathic concern (β ± SE = 0.7 ± 0.21; *p* = 0.015) and MIRI total scores (β ± SE = 1.1 ± 0.34; *p* = 0.014).

**Discussion:**

Musical experience in earlier life and potentially ongoing regular music listening may ameliorate socio-emotional functioning in bvFTD. Future work in larger cohorts is required to substantiate the robustness of this association, establish its mechanism and evaluate its clinical potential.

## Introduction

While it is well established that musical expertise can shape brain structure and function (1, 2), the potential of music to modify the clinical expression of neurodegenerative brain pathologies has been largely unexplored. Limited evidence suggests that training on a musical instrument may benefit cognitive task performance in domains such as executive function and verbal memory, and may enhance the task-related functional connectivity of neural networks (3–5). Moreover, musicians may have a reduced incidence of dementia (6, 7). However, the mechanism of any protective effect and its disease specificity have not been clarified.

On neurobiological as well as clinical grounds, this issue may be particularly pertinent in the behavioural variant of frontotemporal dementia (bvFTD). This syndrome typically presents with impaired social and emotional awareness, empathy and perspective taking, accompanied by diverse abnormal behaviours including disinhibition, impulsivity, tactlessness, mental rigidity, obsessionality, perseveration and inertia (8). Music processing and social cognition engage common neural mechanisms, and these same mechanisms are targeted early and relatively selectively in bvFTD (9, 10). Long-term musical exposure is associated both with enhanced emotion recognition (11) and increased connectivity and functional integration in the salience network (1), a core target of the pathological process in bvFTD. This is in line with other work suggesting that certain occupational and lifestyle exposures may attenuate the phenotypic impact of bvFTD (12). Further, developmental amusia (‘tone deafness’) is associated with deficits of social signal processing in otherwise cognitively normal adults (13). Taken together, such evidence suggests that musical experience might modulate vulnerability to the clinical expression of bvFTD, putatively via effects on neural network resilience and reserve (12, 14).

Here we addressed whether past musical experience, current musical listening habits and/or musical perceptual skills affect the phenotypic expression of bvFTD. We studied a well-characterised bvFTD cohort, assessing patients musically using a customised caregiver survey and cognitive tests. Based on available evidence (1, 2, 9, 10, 12), we hypothesised that cumulative past musical experience would modulate behavioural symptoms of socio-emotional dysfunction in patients with bvFTD, independently of effects on general executive and other cognitive abilities.

## Methods

### Participants

Fourteen consecutive patients with bvFTD were recruited via a national tertiary referral centre in London, United Kingdom. All fulfilled consensus diagnostic criteria for bvFTD (8), supported by a comprehensive clinical, neuropsychological and behavioural assessment (Table 1) and brain MRI showing a compatible profile of atrophy. Exclusion criteria comprised inability to understand English or give informed consent, or neurological or psychiatric comorbidities. To interpret the clinical and musical profile of the bvFTD cohort, patients were referenced to a historical cohort of 29 demographically similar, healthy older British adults (details in Table 1), who had been assessed on the same musical, behavioural and neuropsychological measures. No patient had a disease-causing genetic mutation. None was known to have had premorbid developmental amusia.

**Table 1:** Characteristics of the bvFTD group relative to healthy older individuals.

	bvFTD	Healthy controls	*p* value
Demographic and clinical			
No. (M:F)	14 (12:2)	29 (15:14)	n.s.
Handedness (R:L)	13:1	24:5	n.s.
Age	65.3 (5.8)	64.3 (5.0)	n.s.
Education (years)	14.6 (2.5)	15.8 (2.6)	n.s.
Peripheral hearing threshold (dB)^a^	28.6 (4.8)	25.4 (8.0)	n.s.
Symptom duration (years)	6.2 (4.2)	n.a.	n.s.
MMSE score	25.4 (3.1)	29.4 (1.0)	**< 0.001**
Musical characteristics			
Past musical experience^b^	1.79 (1.5)	1.83 (1.2)	n.s.
Music listening currently (hours/week)	7.4 (6.7)	7.5 (8.6)	n.s.
BMRQ	25.2 (7.8)	26.2 (4.4)	n.s.
Musical skills^c^			
Pitch change discrimination (/10)	7.4 (2.0)	9.1 (1.2)	**0.001**
Familiar melody recognition (/48)	41.5 (7.9)	46.1 (2.2)	**0.01**
Socio-emotional functioning			
CBI-R	77.5 (33.9)	6.4 (4.3)	**< 0.001**
MIRI total	33.1 (11.9)	62.8 (3.6)	**< 0.001**
MIRI empathetic concern	19.7 (7.8)	33.6 (1.3)	**0.001**
MIRI perspective taking	13.4 (6.0)	29.2 (2.6)	**< 0.001**
General cognitive functions			
*General executive*			
WASI Block Design (/71)	27.2 (14.0)	48.8 (12.1)	**< 0.001**
WASI Matrices (/32)	16.6 (6.9)	26.2 (3.7)	**< 0.001**
Stroop colour naming (s)	48.1 (19.9)	29.4 (5.7)	**< 0.001**
Stroop word reading (s)	29.6 (12.6)	21.8 (4.9)	**0.007**
TMT-A (s)	58.3 (25.3)	29.7 (11.2)	**< 0.001**
TMT-B (s)	139.8 (73.3)	62.5 (20.6)	**< 0.001**
*Working memory*			
WMS-R digit span forward (max)	6.3 (0.9)	7.1 (1.1)	**0.02**
WMS-R digit span reverse (max)	4.5 (1.6)	5.8 (1.1)	**0.002**
*Episodic memory*			
RMT words (/50)	38.0 (7.4)	47.9 (2.3)	**< 0.001**
RMT faces (/50)	34.3 (7.0)	41.1 (6.0)	**0.002**
Camden PAL (/24)	11.8 (6.8)	19.6 (3.9)	**< 0.001**
*Language*			
WASI vocabulary (/80)	47.8 (20.2)	72.2 (3.6)	**< 0.001**
Graded naming test (/30)	16.7 (9.8)	25.1 (2.8)	**< 0.001**
BPVS (/150)	130.6 (31.9)	147.8 (1.8)	**0.006**
Letter fluency (F, 1 min)	8.8 (5.8)	18.5 (5.7)	**< 0.001**
Category fluency (animals, 1 min)	11.9 (5.5)	25.2 (2.6)	**< 0.001**
*Visuoperceptual*			
VOSP Object Decision (/20)	15.9 (3.5)	18.4 (1.8)	**0.003**

The table summarises the demographic, musical, clinical, behavioural and neuropsychological characteristics of the participating bvFTD patients, referenced to an historical cohort of demographically similar, healthy older individuals. Data are presented as mean (standard deviation) values unless otherwise indicated; maximum scores on neuropsychological tests are shown in parentheses. Significant differences between groups (*p* < 0.05) are indicated in bold. ^a^Assessed using pure tone audiometry (details in Supplementary material); ^b^Scored from musical experience survey (see text and Supplementary material—details about musical background and current musical habits for individual bvFTD patients are in Supplementary Table S1); ^c^Assessed using previously described procedures (14) details in Supplementary material. BMRQ, Barcelona music reward questionnaire; BPVS, British picture vocabulary scale; bvFTD, Patient group with behavioural variant frontotemporal dementia; CBI-R, Cambridge behavioural inventory—revised; dB, Decibels; F, Female; L, Left; M, Male; MIRI, Modified interpersonal reactivity index; MMSE, Mini-mental state examination; n.a., Not applicable; n.s., Not significant; PAL, Paired associate learning test; R, Right; RMT, Recognition memory test; s, seconds; TMT, Trail making test (scores based on maximum time achievable, 2.5 min on task A and 5 min on task B); VOSP, Visual object and spatial perception battery; WASI, Wechsler abbreviated scale of intelligence; and WMS, Wechsler memory scale.

The study was approved by UCL/UCLH Joint Research Ethics Committee, and all patients gave informed consent to participate, in line with Declaration of Helsinki guidelines.

### Assessment of musical experience

Patients’ past musical experience and current musical listening habits were assessed using a structured survey (see details in Supplementary material). Past experience of playing an instrument or singing was scored on a five-point scale, ranging from 0 (no past experience) to 4 (United Kingdom grade 7 or 8 qualification on an instrument or for singing). The estimated average number of hours the patient currently spent listening to music each week was also recorded. In addition, we administered the Barcelona Music Reward Questionnaire (BMRQ) (15), an index of pleasure in music. Musical survey information was gathered from and/or endorsed by each patient’s long-term primary caregiver.

### Assessment of musical perceptual skills

To determine patients’ current musical skills, we administered tests assessing discrimination of pitch change direction and familiar melody recognition, using previously described procedures (14) (details in Supplementary material).

### Assessment of behavioural disturbance and socio-emotional functioning

Behavioural changes and socio-emotional cognition were assessed using standardised informant-based questionnaires. Patients’ primary caregivers completed the Cambridge Behavioural Inventory (Revised) (CBI-R) (16), an index of general neurobehavioural and psychiatric symptoms; and the Modified Interpersonal Reactivity Index (MIRI) (17), an index of empathy and perspective taking. Whereas higher CBI-R score reflects more behavioural impairment, higher MIRI score reflects better socio-emotional functioning.

### Statistical analysis

Statistical analyses were performed in R (v4.1.0). Demographic and clinical characteristics were investigated using T-tests and chi-squared tests where appropriate. Associations of past musical experience (survey scores), current music listening (hours spent listening to music each week), and musical test (pitch discrimination, melody recognition) scores with demographic and socio-emotional behavioural measures (separately for each of the subscales of the MIRI) were assessed within the bvFTD group using linear regressions. The analyses were repeated in the healthy control group to investigate the robustness of the associations. In the regression model, we adjusted for age, sex, years of formal education and measures of overall cognitive functioning (Mini-Mental State Examination score) and general executive function (WASI Block Design score). We also assessed the associations of patients’ musical experience with each of the neuropsychological tests (as a control for nonspecific disease effects). Results were plotted using lines of best fit based on Akaike Information Criterion (AIC) and Bayesian Information Criterion (BIC). A threshold *p* < 0.05 was accepted as the criterion for statistical significance in all analyses.

## Results

### Characteristics of the bvFTD group

Demographic, musical, clinical, neuropsychological and behavioural characteristics of the bvFTD group are summarised in Table 1 (referenced to an historical cohort of healthy older individuals). Patients exhibited typical clinical and neuropsychological profiles of bvFTD.

Information about musical background and current musical listening habits for individual bvFTD patients are presented in Supplementary Table S1. While most patients had received at least some musical training, individual patients ranged widely in past musical experience: one had been a professional musician and four had never played an instrument. Caregiver survey responses further indicated considerable variation of current music listening within the bvFTD group, including patients who had developed musicophilia and music aversion as part of their illness. Overall, however, the patient group had similar musical characteristics to the historical healthy control cohort (Table 1). Patients as a group performed significantly worse than healthy controls on tests assessing current musical (pitch discrimination and melody recognition) skills.

### Associations with past musical experience in bvFTD

**Figure 1:**
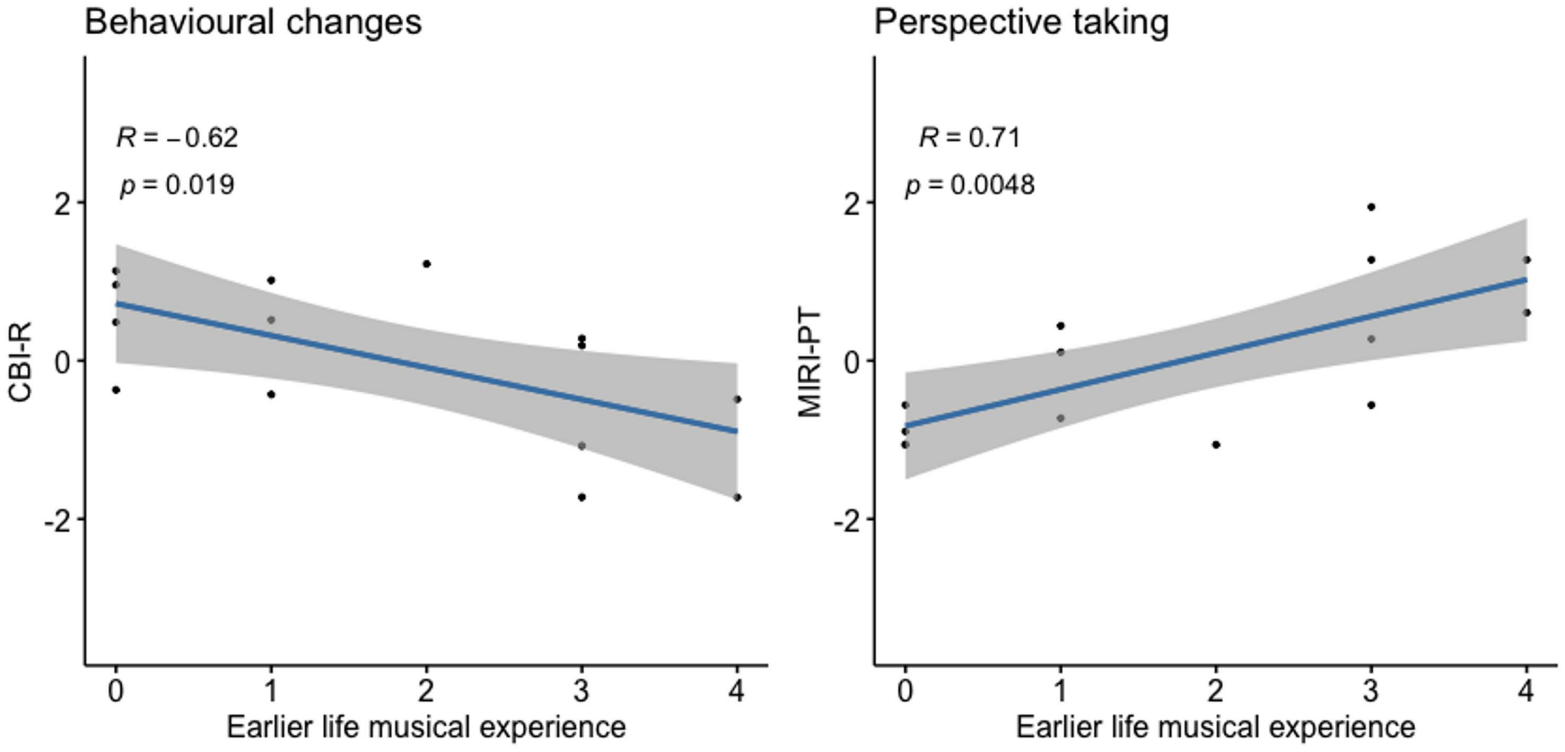
Association of past musical experience with socio-emotional functioning in bvFTD. Scores of individual bvFTD patients on standard measures of daily life socio-emotional functioning vs. their scores on our earlier life musical experience survey (graded between 0 and 4; see text and Supplementary material) are plotted, with lines of best fit and 95% confidence envelopes. CBI-R and MIRI-PT scores are *z*-transformed; Pearson correlations with corresponding *p* values are indicated on the plots. bvFTD, Patient group with behavioural variant frontotemporal dementia; CBI-R, Cambridge behavioural inventory—revised; and MIRI-PT, modified interpersonal reactivity index—perspective taking.

Correlations of past musical experience with other cognitive and behavioural measures in the bvFTD group are presented in Figure 1 and Table 2. Within the bvFTD group, a higher past musical experience score was associated with significantly lower CBI-R score (β ± SE = −17.2 ± 5.2; 95% CI [−5.2, −29.3]; BIC/AIC = 134/129; *R*^2^ = 0.79; *p* = 0.01) and higher MIRI perspective-taking subscore (β ± SE = 2.8 ± 1.1; 95% CI [0.3, 5.3]; BIC/AIC = 90/85; *R*^2^ = 0.71; *p* = 0.03). Greater past musical experience was also significantly associated with higher number of hours currently spent listening to music each week (β ± SE = 4.02 ± 1.55; 95% CI [0.5, 7.6]; BIC/AIC = 100/95; *R*^2^ = 0.52; *p* = 0.03), and with better performance on the pitch discrimination test (β ± SE = 1.2 ± 0.27; 95% CI [0.6, 1.9]; BIC/AIC = 51/47; *R*^2^ = 0.82; *p* = 0.0019). Past musical experience was not associated with performance on general neuropsychological tests (all *p* > 0.05). In particular, there was no association with performance on standard tests of executive function (Table 2). When repeating regression analyses in the normal older control group, no significant associations were found (all *p* > 0.05, Supplementary Table S2).

**Table 2:** Associations of past musical experience and current musical listening habits and skills with socio-emotional and neuropsychological measures in bvFTD.

	Past musical experience	Pitch discrimination	Familiar melody recognition	Music listening (hours/week)
Music listening habits and skills				
Pitch discrimination	**1.24 (0.27)** ^**^			
Familiar melody recognition	5.82 (4.27)	3.15 (3.25)		
Music listening (hours/week)	**4.02 (1.55)** ^*^	2.49 (1.11)	0.33 (0.17)	
BMRQ	3.53 (1.29).	1.83 (1.35)	0.51 (0.73)	0.60 (0.35)
Demographic and clinical				
Education years	0.96 (0.43).	0.052 (0.47)	0.05 (0.06)	0.04 (0.12)
MMSE score	0.28 (0.78)	−0.18 (0.54)	−0.02 (0.08)	−0.15 (0.13)
Socio-emotional functioning				
CBI-R	**−17.24 (5.22)** ^*^	−10.37 (4.64).	−0.33 (0.83)	−2.23 (1.33)
MIRI total	4.97 (2.16)	2.59 (1.81)	0.24 (0.26)	**1.10 (0.34)** ^*^
MIRI empathetic concern	2.19 (1.49)	1.03 (1.21)	0.08 (0.17)	**0.67 (0.21)** ^*^
MIRI perspective taking	**2.78 (1.09)** ^*^	1.56 (0.87)	0.16 (0.14)	0.42 (0.22).
General cognitive functions				
*General executive*				
WASI block design (/71)	0.61 (3.57)	2.76 (1.77)	0.14 (0.29)	0.13 (0.60)
WASI matrices (/32)	−1.44 (1.30)	−1.01 (0.97)	0.11 (0.13)	−0.24 (0.26)
Stroop colour naming (s)	−2.92 (4.18)	−2.90 (2.71)	−0.64 (0.75)	−0.66 (0.75)
Stroop word reading (s)	−2.18 (3.78)	−0.035 (0.98)	−0.15 (0.34)	−1.43 (0.93)
TMT-A (s)	−0.70 (4.53)	−2.11 (2.95)	−0.97 (0.64)	−1.02 (0.70)
TMT-B (s)	23.12 (17.35)	8.02 (9.83)	−2.95 (3.39)	2.91 (2.77)
*Working memory*				
WMS-R digit span forward (max)	0.47 (0.24).	0.35 (0.14)*	0.01 (0.02)	0.09 (0.04)
WMS-R digit span reverse (max)	0.14 (0.34)	0.05 (0.23)	−0.0041 (0.029)	0.028 (0.06)
*Episodic memory*				
RMT words (/50)	−0.37 (1.68)	0.22 (1.23)	0.22 (0.12)	0.09 (0.32)
RMT faces (/50)	1.32 (1.49)	1.25 (1.04)	0.17 (0.13)	0.15 (0.29)
Camden PAL (/24)	1.53 (1.62)	1.02 (1.43)	**0.49 (0.10)** ^*^	0.16 (0.42)
*Language*				
WASI vocabulary (/80)	1.71 (3.13)	0.96 (2.07)	0.33 (0.28)	0.60 (0.50)
Graded naming test (/30)	−1.32 (2.36)	−0.5039 (1.26)	−0.05 (0.17)	−0.18 (0.32)
BPVS (/150)	9.20 (8.62)	7.22 (3.70).	1.13 (0.53).	2.04 (0.91).
Letter fluency (F, 1 min)	−0.02 (1.16)	−0.34 (0.99)	0.14 (0.28)	0.04 (0.27)
Category fluency (animals, 1 min)	−0.87 (1.03)	−1.40 (0.69).	0.38 (0.17).	−0.02 (0.24)
*Visuoperceptual*				
VOSP Object Decision (/20)	−0.01 (1.33)	−1.04 (0.83)	0.30 (0.17)	−0.28 (0.26)

The table shows correlations between past musical experience (score on the earlier life musical experience survey), current music listening habits and perceptual skills and general cognitive and behavioural measures within the bvFTD group. Analyses are adjusted for age, sex, years of formal education and measures of overall cognitive functioning (mini-mental state examination score) and general executive function (WASI block design score). Data are presented as β (SE). Significant correlations (*p* < 0.05) are in bold; ^*^p ≤ 0.05; ^**^p ≤ 0.01; BMRQ, Barcelona music reward questionnaire; BPVS, British picture vocabulary scale; CBI-R, Cambridge behavioural inventory—revised; MIRI, Modified interpersonal reactivity index; PAL, Paired associate learning test; RMT, Recognition memory test; s, seconds; TMT, Trail making test (scores based on maximum time achievable, 2.5 min on task A and 5 min on task B); VOSP, Visual object and spatial perception battery; WASI, Wechsler abbreviated scale of intelligence; and WMS, Wechsler memory scale.

### Associations with current music listening habits and skills in bvFTD

**Figure 2:**
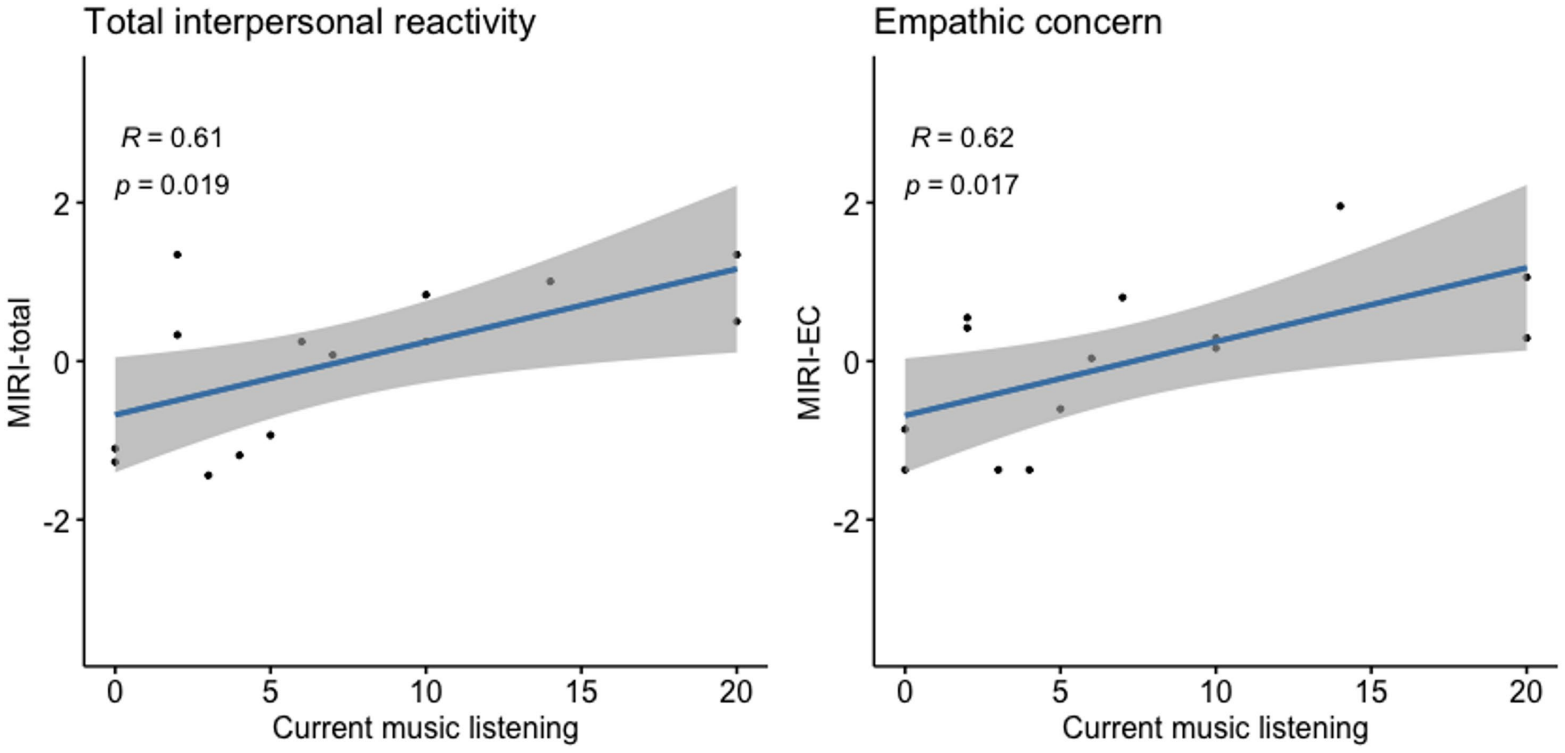
Association of current music listening with socio-emotional functioning in bvFTD. Scores of individual bvFTD patients on standard measures of daily life socio-emotional functioning versus their scores on our earlier life musical experience survey (graded between 0 and 4; see text and Supplementary material) are plotted, with lines of best fit and 95% confidence envelopes. MIRI-total and MIRI-EC scores are *z*-transformed; Pearson correlations with corresponding *p* values are indicated on the plots. bvFTD, Patient group with behavioural variant frontotemporal dementia; MIRI-EC, Modified interpersonal reactivity index—empathic concern; and MIRI-total, Modified interpersonal reactivity index—total score.

Current music listening (higher number of hours per week) was significantly associated with higher MIRI empathic concern (β ± SE = 0.7 ± 0.21; BIC/AIC = 92/87; 95% CI [0.2, 1.2]; *R*^2^ = 0.84; *p* = 0.015) and MIRI total scores (β ± SE = 1.1 ± 0.34; 95% CI [0.3, 1.9]; BIC/AIC 105/100; *R*^2^ = 0.81; *p* = 0.014; Figure 2) but not with performance on general neuropsychological tests (all *p* > 0.05). Scores on tests assessing musical skills (pitch discrimination, melody recognition) were not significantly associated with CBI-R or MIRI scores in the bvFTD cohort (all p > 0.05). Pitch discrimination scores were significantly associated with a standard measure of auditory working memory, digit span forward (β ± SE = 0.35 ± 0.14; 95% CI [0.01, 0.7]; BIC/AIC = 44/38; *R*^2^ = 0.61; *p* = 0.045), while melody recognition performance was significantly associated only with a measure of episodic memory, paired associate learning (β ± SE = 0.49 ± 0.10; 95% CI [0.05, 0.9]; BIC/AIC = 37/36; *R*^2^ = 0.98; *p* = 0.042). When repeating regression analyses in the normal older control group, no significant associations were found (all *p* > 0.05, Supplementary Table S2).

## Discussion

In this preliminary study, we have shown that greater earlier life musical experience and current regular listening to music (but not musical processing skills) correlate with lower levels of socio-emotional behavioural disturbance in a group of patients with bvFTD, after taking potentially confounding demographic, clinical and cognitive variables into account. This effect was not found in other, extra-musical cognitive domains, suggesting it may be specific for socio-emotional function, and in particular ‘theory of mind’ or awareness of others’ mental states (the MIRI domains of perspective-taking and empathic concern). We speculate that earlier life musical experience may be protective in bvFTD, perhaps by promoting resilience in brain circuits mediating behavioural regulation and theory of mind (1, 13): both key elements of interpersonal conduct in daily life.

These findings corroborate emerging evidence that musical training and other specific competencies in earlier life may influence the phenotypic expression of dementia (7, 12) and further align with previous work suggesting that music plays a role in building social cognitive skills during brain development (13). Further, we found that more regular current music listening was associated with higher empathic concern and overall interpersonal reactivity. These findings underline the potential value of continuing music listening to enhance socio-emotional engagement in dementia (18). Future studies should additionally investigate the effects of continued musical performance, engaging in inter-personal musical activities and active music-based interventions on socio-emotional functioning in bvFTD, and extend the investigation both in healthy older individuals and in patients with different dementia syndromes. It should be noted, however, that current music listening was here also positively correlated with past musical experience, raising the possibility that earlier life musical training primes later life listening habits, and thereby drives any ongoing beneficial effect from music listening. On the other hand, socio-emotional functioning did not correlate with level of musical perceptual skill. The interplay of these factors is not resolved by the current study. When repeating the regression analyses in the healthy older control group we found no associations of musical experience with socio-emotional functioning. This might reflect the limited range and sensitivity of socio-emotional measures in cognitively healthy people, and replication in larger cohorts are required for robust associations.

There are several important caveats on the interpretation of our findings. This is a small patient sample, and musical characteristics were assessed in part from potentially biassed or inaccurate caregiver reports. Furthermore, other non-musical factors (e.g., ethnic, cultural and linguistic) may influence behavioural outcomes in bvFTD (19), and were not accounted for here. Our results await prospective replication in larger, more diverse cohorts, with more detailed analysis of potentially relevant (or confounding) musical as well as demographic and clinical variables. There is a need to disambiguate the effects of past musical experience from current musical listening habits, particularly in planning musical interventions for people with established dementia. Notwithstanding the small cohort size, it is noteworthy that the inverse associations here between past musical experience, current music listening and socio-emotional dysfunction in bvFTD were robust to adjustments for potentially confounding executive and general cognitive factors. Moreover, none of the general cognitive measures sampled showed a significant association with past musical exposure or current music listening (Table 2). Earlier life musical exposure is, however, likely to correlate with a range of other non-musical characteristics and exposures that remain to be defined; nor is musical training (the main proxy for musical experience here) a prerequisite for musicality. More fundamentally, association does not establish causation: people with innately more resilient behavioural regulation and social cognition circuitry might be more likely to learn a musical instrument, or continue listening regularly to music. The socio-emotional benefits of musical training remain poorly understood, and causal relations could be further investigated with alternative indices of musicality and prospective longitudinal studies (11, 20).

Acknowledging these caveats, we hope that our findings will motivate further studies of musicality as a possible modulatory factor in FTD and other dementias—to clarify the nature of the modulatory effect, to establish its neural basis using functional and connectivity-based neuroimaging, and to explore its clinical potential. Analogous with the proposed link between developmental language disorders and primary progressive aphasia (21), one might ask whether the common complaint of developmental amusia (13) confers an increased vulnerability to FTD—and whether cumulative past musical engagement and indeed, continuing regular music listening or music-based behavioural interventions might ameliorate the impact of the disease.

## Data availability statement

The data analysed in this study are subject to the following licences/restrictions: we used patient datasets of the Dementia Research Centre. Requests to access these datasets should be directed to JW, jason.warren@ucl.ac.uk.

## Ethics statement

The study was approved by UCL/UCLH Joint Research Ethics Committee, reference number 06N032. The studies were conducted in accordance with the local legislation and institutional requirements. The participants provided their written informed consent to participate in this study.

## Author contributions

JH: Conceptualization, Formal Analysis, Investigation, Software, Visualization, Writing – original draft, Writing – review & editing. EB: Data curation, Writing – review & editing. CA: Data curation, Writing – review & editing. JJ: Data curation, Writing – review & editing. BL: Writing – review & editing. LC: Writing – review & editing. M-CR-K: Data curation, Writing – review & editing. CH: Writing – review & editing. BT: Writing – review & editing. YP: Writing – review & editing. JW: Conceptualization, Funding acquisition, Methodology, Project administration, Supervision, Writing – review & editing.
