# Supplementary material for: Musical experience influences socio-emotional functioning in behavioural variant frontotemporal dementia
Source: Front Neurol. 2024 Jan 24;15:1341661. doi: 10.3389/fneur.2024.1341661 (PMC10851745; doi:10.3389/fneur.2024.1341661)
Supplement: Supplementary file 1 [file Data_Sheet_1.pdf]

Supplementary material: Musical experience influences socio-emotional functioning in behavioural variant frontotemporal dementia, by J.J. van 't Hooft et al

Scoring of earlier life musical experience

The following scoring procedure was adopted to generate a score for each participant's earlier life musical experience. The musical grades are part of recognized practical musical exams of the United Kingdom, with 8 being the maximum grade (https://gb.abrsm.org/en/our-exams/what-is-a-graded-music-exam/).

Score 0: never played an instrument or engaged in singing performance, whether or not formal lessons

Score 1: played an instrument or engaged in singing performance for less than a year, whether or not formal lessons

Score 2: more than 2 years' experience playing an instrument or singing performance but did not obtain any UK grade qualifications

Score 3: obtained UK grade 3, 4, 5 or 6 qualification on an instrument or for singing **or** at least 10 years' experience playing an instrument or singing performance without obtaining a grade qualification

Score 4: obtained UK grade 7 or 8 qualification on an instrument or for singing

Assessment of peripheral hearing function

We adopted the procedure described by Benhamou and colleagues (2021). Using an Otovation Roto® audiometer (https:/www.auditdata.com/) with a single TDH-39P 10-ohm Telephonics® earphone (www.telephonics.com) in a quiet room, steady tones of 500, 1000, 2000, 4000 and 6000Hz were presented separately to each of the participant's ears, over ascending intensity levels commencing at 20 dB HL (decibel hearing level). At each frequency, the participant indicated (verbally or by gesture) when they first heard a noise. If the participant was unable to hear the tone, the level was increased in 5 dB increments (maximum 70dB HL). This procedure was repeated three times to establish the mean threshold at that frequency. For each participant, a composite score was created by calculating the mean threshold across all frequencies in the best ear.

Assessment of pitch change direction processing

To provide a measure of elementary pitch pattern processing relevant to perception of melodies, we adopted the procedure described by Benhamou and colleagues (2021). We assessed each participant's ability to discriminate pitch direction changes in sequentially presented note pairs; the notes comprising each pair differed in pitch by one to five semitones. Individual notes comprising each pair had piano timbre and duration 1 second with inter-note gap 1 second. Ten trials (pairs) were presented and the direction of the pitch shift between notes in each pair was varied randomly across trials (five ascending, five descending). The task on each trial was to decide if the second note of the pair was 'higher' or 'lower' than the first note.

Assessment of familiar melody recognition

To provide a measure of familiar melody recognition (musical semantic memory), we adopted another test procedure described by Benhamou and colleagues (2021). Each participant was asked to listen to 24 short melodies (all previously rated as highly familiar to older British people), randomly interspersed with 24 unfamiliar (newly composed) melodies, closely matched to the familiar melody set for loudness, duration, key, pitch range and tempo. The task on each trial was to indicate whether or not the melody was a well-known tune.

Table S1. Summary of earlier life musical experience and current musical habits among bvFTD patients

Case	Musical survey responses	Experience score	Current listening (hrs/week)	Comments
1	No musical training<br>Listens to music every day, especially rock, some pop / jazz, less classical but no real change since diagnosis	0	3	
2	Sang in choir when young but no formal musical training.<br>Does not purposely listen to music at the moment	1	0	Music aversion
3	Played clarinet for a few months<br>Listens to [BBC] Radio 1 - pop/rock, and really likes classical music on [BBC] Radio 4	1	2	
4	Self-taught trumpet, guitar, singing for 10 years<br>Still plays guitar at home, listens to music, goes to concerts, festivals - classical, rock	3	4	
5	Music lessons in school, played guitar<br>Does not play anymore but listens to music every day - jazz, classical, rock, pop	3	10	
6	No musical training.<br>Listens to music every day - Reggae, Sex Pistols, The Clash	0	14	Musicophilia
7	Probably never had musical training when younger<br>Currently doing Youtube tutorial to learn violin and practices violin every day; listens to music regularly, mostly rock	1	6	
8	Musical training in choir, piano and organ, and choral professional<br>Still plays piano; listens to concerts, especially classical	4	10	
9	No musical training<br>Does not listen to music anymore, used to listen regularly	0	0	Music aversion
10	Piano grade 8<br>Still plays every day (classical pieces); listens to Classic FM every day	4	20	Musicophilia
11	No musical training, never played an instrument<br>Listens to Indian music at home	0	7	
12	Self-taught guitar<br>Still plays guitar around an hour each week; listens to music every day – Smooth Radio, rock, soul	2	5	
13	Played piano aged 11 for a few months, guitar for 3 years, no grade<br>Now sings in choir each week; listens to music every day, classical music, pop / rock	3	2	
14	Training on violin, in choir, played violin for 10 years.<br>Currently sings every week; listens to music every day, mainly classical	3	20	Musicophilia

The table summarises the musical background and current listening and/or performance habits of each of the participating bvFTD patients, as reported and/or endorsed by primary caregivers. Scores for past musical training and other musical performance experience (see above for details of scoring procedure) and estimated average hours spent listening to music each week are indicated. Patients were classified as having musicophilia if they had a clear increased liking for music with an obsessional flavour since onset of their illness.

Table S2. Associations of past musical experience and current musical listening habits and skills with socio-emotional tests in the healthy control group

	Past musical experience	Pitch discrimination	Familiar melody recognition	Music listening (hours / week)
CBI-R	-1.06 (1.21)	-3.5 (1.88)	-0.8471	-0.14 (0.31)
MIRI total	1.44 (1.30)	3.25 (1.33) .	2.96 (0.73)	0.40 (0.13) .
MIRI empathetic concern	0.61 (0.28)	0.88 (0.70)	1.69 (1.47)	0.13 (0.07)
MIRI perspective taking	0.83 (1.06)	2.39 (0.09) .	1.27 (0.73)	0.27 (0.11) .

The table shows correlations between past musical experience (score on the earlier life musical experience survey), current music listening habits and perceptual skills and behavioural measures within the healthy control group. Analyses are adjusted for age, sex, years of formal education and measures of overall cognitive functioning (Mini-Mental State Examination score) and general executive function (WASI Block Design score). Data are presented as β (SE). Significant correlations ($p<0.05$) are in bold; · $p\leq0.1$; CBI-R, Cambridge Behavioural Inventory - Revised; MIRI, Modified Interpersonal Reactivity Index
